# Plasma Biomarkers of Risk of Tuberculosis Recurrence in HIV Co-Infected Patients From South Africa

**DOI:** 10.3389/fimmu.2021.631094

**Published:** 2021-03-25

**Authors:** Kimesha Pillay, Lara Lewis, Santhuri Rambaran, Nonhlanhla Yende-Zuma, Derseree Archary, Santhanalakshmi Gengiah, Dhineshree Govender, Razia Hassan-Moosa, Natasha Samsunder, Salim S. Abdool Karim, Lyle R. McKinnon, Nesri Padayatchi, Kogieleum Naidoo, Aida Sivro

**Affiliations:** ^1^ Centre for the AIDS Programme of Research in South Africa (CAPRISA), Durban, South Africa; ^2^ MRC-CAPRISA HIV-TB Pathogenesis and Treatment Research Unit, Doris Duke Medical Research Institute, University of KwaZulu-Natal, Durban, South Africa; ^3^ Department of Medical Microbiology, University of KwaZulu-Natal, Durban, South Africa; ^4^ Department of Epidemiology, Columbia University, New York City, NY, United States; ^5^ Department of Medical Microbiology and Infectious Diseases, University of Manitoba, Winnipeg, MB, Canada; ^6^ Department of Medical Microbiology, University of Nairobi, Nairobi, Kenya

**Keywords:** inflammation, HIV, microbial translocation, antiretroviral (ARV) therapy, tuberculosis - pulmonary

## Abstract

There is an urgent need to identify immunological markers of tuberculosis (TB) risk in HIV co-infected individuals. Previously we have shown that TB recurrence in HIV co-infected individuals on ART was associated with markers of systemic inflammation (IL-6, IL1β and IL-1Rα). Here we examined the effect of additional acute inflammation and microbial translocation marker expression on risk of TB recurrence. Stored plasma samples were drawn from the TB Recurrence upon Treatment with HAART (TRuTH) study, in which individuals with previously treated pulmonary TB were screened for recurrence quarterly for up to 4 years. Recurrent TB cases (n = 37) were matched to controls (n = 102) by original trial study arm assignment and ART start date. Additional subsets of HIV infected (n = 41) and HIV uninfected (n = 37) individuals from Improving Recurrence Success (IMPRESS) study were sampled at active TB and post successful treatment completion. Plasma concentrations of soluble adhesion molecules (sMAdCAM, sICAM and sVCAM), lipopolysaccharide binding protein (LBP) and transforming growth factor-beta (TGF-β1, TGF-β2, TGF-β3) were measured by multiplex immunoassays and ELISA. Cytokine data was square root transformed in order to reduce variability. Multivariable analysis adjusted for a number of potential confounders measured at sample time-point: age, BMI, CD4 count, viral load (VL) and measured at baseline: presence or absence of lung cavities, previous history of TB, and WHO disease stage (4 vs 3). The following analytes were associated with increased risk of TB recurrence in the multivariable model: sICAM (aOR 1.06, 95% CI: 1.02-1.12, p = 0.009), LBP (aOR 8.78, 95% CI: 1.23-62.66, p = 0.030) and TGF-β3 (aOR 1.44, 95% CI 1.01-2.05, p = 0.044). Additionally, we observed a positive correlation between LBP and sICAM (r= 0.347, p<0.0001), and LBP and IL-6, identified to be one of the strongest predictors of TB risk in our previous study (r=0.623, p=0.03). These data show that increased risk of TB recurrence in HIV infected individuals on ART is likely associated with HIV mediated translocation of microbial products and the resulting chronic immune activation.

## Introduction

Despite being a preventable and treatable disease, tuberculosis (TB) is currently one of the top ten causes of mortality globally and ranks above human immunodeficiency virus/acquired immunodeficiency syndrome (HIV/AIDS) as the primary cause of mortality from an infectious agent (1). Africa accounted for 25% of new TB cases in 2019, partly driven by the overlapping HIV epidemic. The risk of developing active TB disease in people living with HIV (PLHIV) is 18 (2–8) times higher compared to HIV uninfected individuals (1). There is a dire need to improve our ability to predict those most at risk for TB disease progression and poor TB treatment response especially among HIV infected patients to enable early implementation of mitigating clinical and public health measures.

The lack of reliable biomarkers of TB risk and treatment response has hindered TB management and drug development. Well defined correlates of TB risk and protection could facilitate rapid screening of new prevention methods and could improve diagnosis of active disease thereby slowing down transmission (9). TB biomarkers are needed partly because of inability to detect the bacterium or its products in easily accessible patient samples. The current diagnosis of active disease and monitoring of response to TB treatment relies on sputum samples, whose volume and quality vary during the course of the disease. Blood-based biomarkers would be advantageous for several reasons, mainly due to relative ease of sample collection, reduced transmission risk, and the ability to measure multiple biomarkers at the same time therefore improving the predictive power of the test (10).

Susceptibility to both HIV and TB infections as well as the course and outcome of the disease are affected by the inflammation induced changes in cytokine/chemokine expression (10–15). Inflammatory responses to both HIV and TB act similarly, whereby the initial response is needed to prevent and contain the infection. However, if left uncontrolled, this inflammatory response can lead to immune mediated pathology (16, 17). HIV mediated translocation of microbial products and the resulting chronic immune activation are known to increase the risk of opportunistic infections, including TB (14). On the other side, both latent and active TB have been shown to exacerbate immune activation in individuals co-infected with HIV (18) potentially contributing to faster HIV disease progression.

Utilizing specimens from the TB Recurrence upon Treatment with HAART (TRuTH) study we have previously identified several inflammatory markers of risk of TB recurrence (IL-6, IL-1β and IL-1Rα) and protection (IFN- β) in HIV co-infected individuals on antiretroviral therapy (ART) (11). Macaque models of TB/simian immunodeficiency virus (SIV) coinfection have demonstrated that SIV-mediated chronic immune activation was the likely driver of reactivation of latent TB infection (14). As the chronic inflammation during HIV/SIV infection is driven by HIV/SIV-mediated gastrointestinal damage and leakage of microbial products we wanted to assess the effect of lipopolysaccharide binding protein (LBP), as a surrogate marker of systemic LPS exposure (19, 20), on risk of TB recurrence in TB-HIV co-infected patients. We additionally expanded our initial observations and examined three adhesion molecules: soluble intracellular adhesion molecule (sICAM), soluble vascular cell adhesion molecule (sVCAM) and soluble mucosal addressin cell adhesion molecule (sMAdCAM) for their roles in inflammation and cell recruitment to the mucosal tissues (21); and three isoforms of the transforming growth factor – beta family (TGF-β1, 2 and 3) for their roles in inflammation, immunoregulation and mucosal barrier repair (2, 3). The effect of the measured analytes on the risk of TB recurrence was done using specimens from the TB Recurrence upon Treatment with HAART (TRuTH) study, in which HIV infected individuals with previously treated pulmonary TB were screened for recurrence quarterly for up to 4 years. Additional cohort (Improving Recurrence Success, IMPRESS) was used to assess the effect of active TB and successful TB treatment completion on the expression of the measured analytes in both HIV infected and uninfected individuals.

## Methods

### Study Participants

Informed written consent for study enrolment and sample storage for future analysis was obtained from all study participants. The University of KwaZulu-Natal’s Biomedical Research Ethics Committee (BE659/17) approved this study. We analyzed stored plasma specimens from two Centre for the AIDS Programme of Research in South Africa (CAPRISA) study cohorts: the 005 TRuTH and CAPRISA 011 IMPRESS. All study participants were recruited and treated at the urban CAPRISA eThekwini Research Clinic in KwaZulu-Natal, South Africa.

The CAPRISA 005 TRuTH observational cohort study investigated the rate of TB recurrence in ART treated adults following completion of treatment for pulmonary TB (Clinical trial NCT 01539005). These participants were previously enrolled in the CAPRISA 003 SAPiT trial (4), which investigated timing of antiretroviral (ART) initiation during pulmonary TB treatment. Participants that entered TRuTH had a confirmatory negative status for TB upon completion of TB treatment. While retained on ART, participants were screened four times a year for a maximum of 4 years for TB recurrence. We conducted a nested case-control study using TRuTH stored samples, where cases, defined by TB recurrence that had an available pre-recurrence sample, were matched on ART start date in 1:3 ratio to controls, defined as those participants with no TB recurrence during follow-up. Cases were sampled at a minimum of 3 and maximum of 9 months prior to TB recurrence, and controls sampled at comparable time points to minimize the difference in sample cryopreservation length between groups. A subset of cases was followed longitudinally at additional time points: Recurrence/TB (2-month window before or after TB recurrence) and cure/post TB (capturing a 2 month-window before and 3 month- window after recurrent TB treatment completion date).

The CAPRISA 011 IMPRESS open-label randomized controlled study (Clinical trials NCT 02114684), compared TB treatment outcomes among previously treated TB patients receiving an interventional Moxifloxacin containing TB regimen compared to standard of care for treatment of smear positive pulmonary TB (5). This study enrolled men and women who were HIV infected and uninfected, >18 years of age, with a documented previous history of TB and current sputum smear positive *Mycobacterium tuberculosis* (*M.tb)* infection. Patients were stratified by HIV status and randomized into the intervention and control arm. To analyze the effect of TB treatment completion on the expression of measured analytes (MAdCAM, ICAM, VCAM, LBP, TGF-β1, TGF-β2 and TGF-β3), a subset of HIV infected and HIV uninfected men and women were sampled at two time-points: active TB disease and post successful TB treatment completion (Cure). No matching was performed.

### Sample Collection and Processing

Acid citrate dextrose (ACD) tubes were used to collect peripheral blood. The blood was centrifuged for 10 minutes at 1600rpm, the plasma was retrieved and cryopreserved for future analysis at -80°C.

### Soluble Analyte Measurement

Plasma levels of sICAM and sVCAM where simultaneously quantified using the Milliplex MAP Human Sepsis kit (Millipore Corporation, St. Charles, MO). Plasma levels of TGF-β1, TGF -β2 and TGF -β3 were quantified using the Transforming Growth factor-beta kit (Bio-Rad, Laboratories Inc., Hercules, CA). All multiplex assays were analyzed on the Bio-Plex ™ 200 system. Soluble LBP (sLBP) plasma levels were quantified using the Human Lipopolysaccharide Binding Protein ELISA (R&D Systems Inc., Minneapolis, MN). Soluble MAdCAM-1 (sMAdCAM-1) levels where quantified using the Hycult Biotech HK337 Human sMAdCAM-1 ELISA kit (Hycult, USA). ELISAs were analyzed using the VersaMax™ ELISA Microplate Reader with SoftMax^®^ Pro Software. All assays were performed as per the manufacturer’s instructions. Briefly, following sample dilutions were used: sMAdCAM-1 (1:10), TFG- β1/2/3 (1:16), sLBP (undiluted), sICAM/sVCAM (1:40). All samples measured for sMAdCAM, sICAM, sVCAM and LBP produced a value within the range of the standard curve (100% detectability). Samples measured for TGF-β1, TGF-β2 and TGF-β3 had high levels of detectability, where TGF-β1 was 97.92% and TGF-β2 and TGF-β3 were 89.06% detectable. The samples with values below the range of the standard curve were assigned the value half of the limit of detection (LOD/2). LODs for the measured analytes were as follows: sMAdCAM-1 (0.41 ng/ml), TFG- β1 (3.9 pg/ml), TFG- β2 (1.9 pg/ml), TFG- β3 (0.5 pg/ml), sLBP (1.5 ng/ml), sICAM (17.7 pg/ml), sVCAM (10.7 pg/ml).

### Statistical Analysis

All specimens were analyzed blinded to the clinical status, with longitudinal samples analyzed on the same plate. GraphPad Prism version 8.3.1 (GraphPad software, La Jolla, CA), SPSS version 24 and SAS version 9.4 were used to perform statistical analysis.

For comparison of baseline characteristics of TRuTH samples between cases and controls, the Wilcoxon signed rank and the McNemar tests were used. Cytokine data was square root transformed in order to reduce the variability, a phenomenon that is known to affect most cytokine measurements. To determine if measured plasma analytes had an effect on the TB recurrence, we conducted a univariable and multivariable conditional logistic regression. Multivariable analysis adjusted for a number of potential confounders measured at sample time-point: age, body mass index (BMI), CD4 count, viral load (VL) and measured at baseline: presence or absence of lung cavities, previous history of TB, and World Health Organization (WHO) disease stage (4 vs 3). Longitudinal samples were compared using paired t-test or Wilcoxon signed rank test. Correlations between analytes were determined using Spearman correlation. The Benjamini-Hochberg method was applied to control for the false discovery rate (FDR), Q:5%.

Baseline and follow-up characteristics in IMPRESS were summarized using percentages and frequencies for categorical variables and median and interquartile range (IQR) values for continuous variables. Changes in measured analytes between active TB and TB cure were analyzed using a paired t-test for normally distributed variables and using a Wilcoxon signed rank test for variables that were not normally distributed. Individuals that failed treatment or for which TB recurrence was observed (n=3) were excluded from the analysis.

## Results

### Study Participants Characteristics and Demographics

*TRuTH cohort*: The final analysis included 139 participants, of which 37 (19 males and 18 females) were cases and 102 (45 males and 57 females) were controls. The median age was 32 years [interquartile range (IQR) 28-35] for cases and 34 years (IQR 28-40) for controls (p=0.025). The median CD4 count was 479.0 cell/mm^3^ (IQR 339.0-834.0) and 458.5 cells/mm^3^ (IQR 360.0 – 631.0, p=0.762), for cases and controls, respectively. Despite ART, some participants had detectable viral loads (VLs): the mean (standard deviation) VL for cases was 1.61 (0.7) log copies/ml and 1.5 (0.6) log copies/ml for controls (p=0.319). A detailed list of the TRuTH cohort characteristics can be found in **Supplementary Table 1**.

*IMPRESS study*: In total, 41 HIV infected and 33 HIV uninfected participants had samples available at both time points. Further 3 HIV infected individuals were excluded from analysis because they failed treatment. Out of the 38 HIV infected participants 16 were females and 22 males and out of the 33 HIV uninfected participants 2 were females and 31 males. The median age at enrolment was 35 (IQR 32-43) years for HIV infected and 33 (IQR 25-49) years for HIV uninfected participants. A detailed list of the IMPRESS cohort characteristics can be found in **Supplementary Table 2**.

### Measured Analytes as Predictors of TB Recurrence in TRuTH

**Figure 1:**
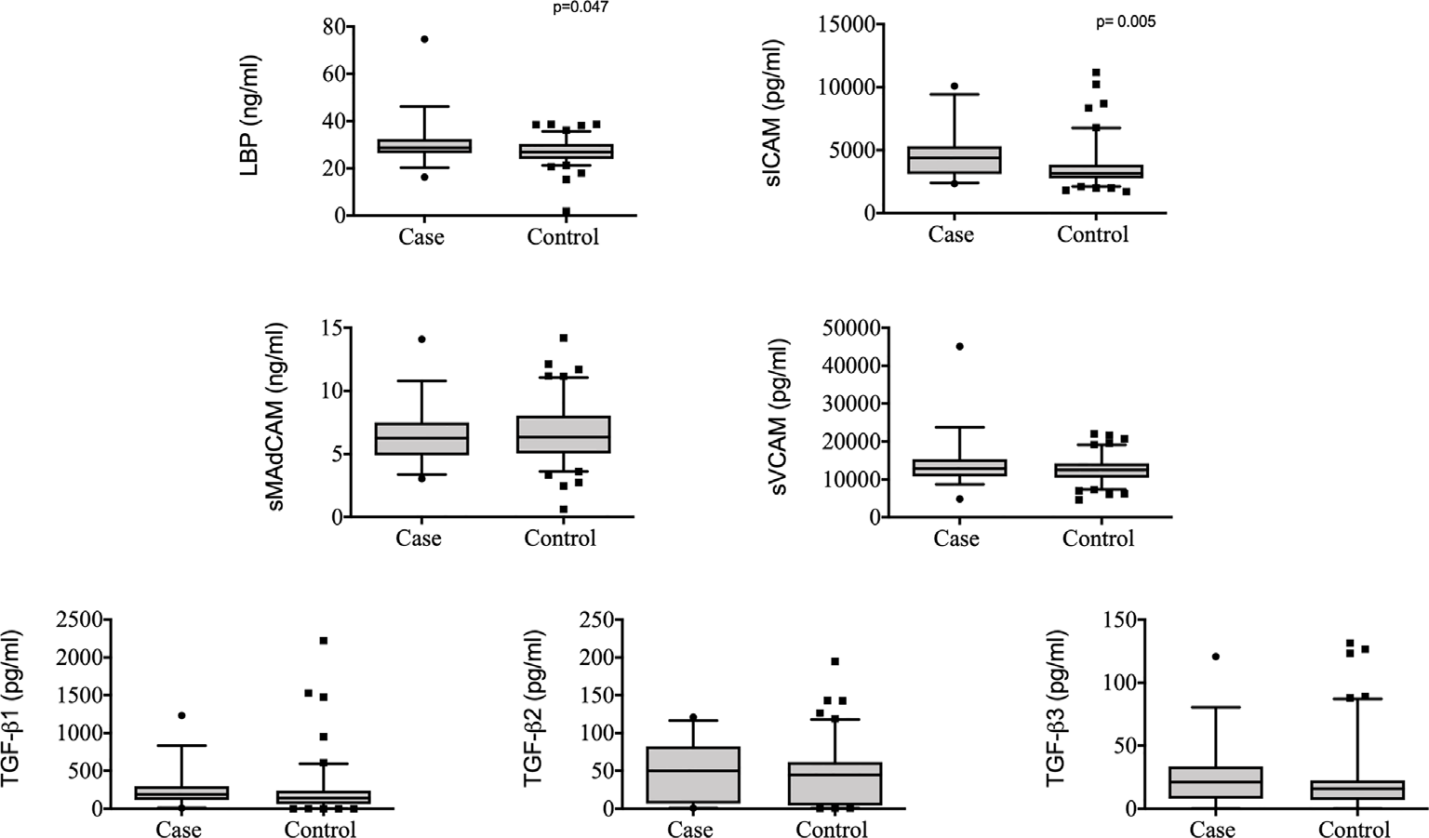
Plasma levels of LBP, sMAdCAM, sICAM, sVCAM, TGF-β1, TGF-β2 and TGF-β3 in controls (n = 103) and cases (n = 37) from TRuTH. Analytes were plotted using Box and Whiskers (5-95%). P-values indicated in the figures are the result of univariable conditional logistic regression (please refer to **Table 1** for full analysis).

To determine if measured plasma analytes had an effect on the rate of TB recurrence, we conducted a univariable and multivariable conditional logistic regression. In the univariable analysis, two analytes were associated with increased odds of TB recurrence: sICAM [Odds Ratio (OR) 1.05, 95% Confidence Interval (CI) 1.01 – 1.08, p = 0.005] and LBP (OR 3.28, 95% CI 1.02 – 10.59, p = 0.047) (**Table 1**, **Figure 1**). These associations remained significant in the multivariable model: sICAM (aOR 1.06, 95% CI 1.02 – 1.12, p = 0.009) and LBP (aOR 8.78, 95% CI 1.23– 62.66, p = 0.030). We also observed a significant correlation between sICAM and LBP expression (r= 0.347, p<0.001, **Supplementary Table 3**). In addition to LBP and sICAM, TGF-β3 (aOR 1.44, 95% CI 1.01 – 2.05, p = 0.044) was significantly associated with increased risk of TB recurrence in the multivariable model (**Table 1**).

**Table 1:** Univariable and multivariable analysis of TRuTH plasma analytes (sMAdCAM, sICAM, sVCAM, LBP, TGF-β1, TGF-β2 and TGF-β3) as biomarkers of TB recurrence.

Cytokine	Univariable		Multivariable^1^	
	OR (95% CI)	p-value	aOR (95% CI)	p-value
sMAdCAM	0.99 (0.39 – 2.52)	0.984	0.94 (0.26 – 3.33)	0.922
sICAM	1.05(1.01 – 1.08)	0.005*	1.06 (1.02 – 1.12)	**0.009**
sVCAM	1.02 (0.99 – 1.04)	0.142	1.02 (0.99 – 1.05)	0.222
LBP	3.28 (1.02 – 10.59)	**0.047**	8.78 (1.23 – 62.66)	**0.030**
TGF- β1	1.05 (0.97 – 1.12)	0.222	1.05 (0.98 – 1.14)	0.179
TGF- β2	1.09 (0.93 – 1.29)	0.274	1.15 (0.93 – 1.43)	0.194
TGF- β3	1.21 (0.95 – 1.55	0.120	1.44 (1.01 – 2.05)	**0.044**

^1^Multivariable analysis adjusted for WHO stage of the disease, BMI, lung cavities, age, CD4 count, VL, gender and previous history of TB. *Statistically significant association after FDR adjustment using 0.05 threshold.

Bold values: p-value < 0.05.

Previously, we measured the plasma expression of 23 plasma cytokines/chemokines to look at their impact on the risk of TB recurrence (11) in the TRuTH cohort. Here we examined if there was a correlation between the expression of 23 measured cytokines/chemokines and sMAdCAM, sICAM, sVCAM, LBP, TGF-β1, TGF-β2 and TGF-β3 in 21 overlapping plasma samples. We observed significant positive correlations between LBP and IL-6 (r=0.623, p=0.003); sVCAM and IL-27 (r=0.438, p=0.047); TGF-β1 and sCD14 (r=0.513 p=0.017); and TGF-β2 with: IL-7 (r=0.481, p=0.027), IL-6 (r=0.438, p=0.047), and sCD14 (r=0.456, p=0.038) (**Supplementary Table 4**).

### Effect of TB Treatment Completion on Plasma Expression of Measured Analytes

A small subset of cases was followed longitudinally to observe the changes in plasma expression of measured analytes (sMAdCAM, sICAM, sVCAM, LBP, TGF-β1, TGF-β2 and TGF-β3) in response to TB treatment completion in the TRuTH cohort (n=14). No significant differences were observed in measured analyte expression between active TB and post TB treatment/Cure time-point (**Supplementary Table 5**).

**Figure 2:**
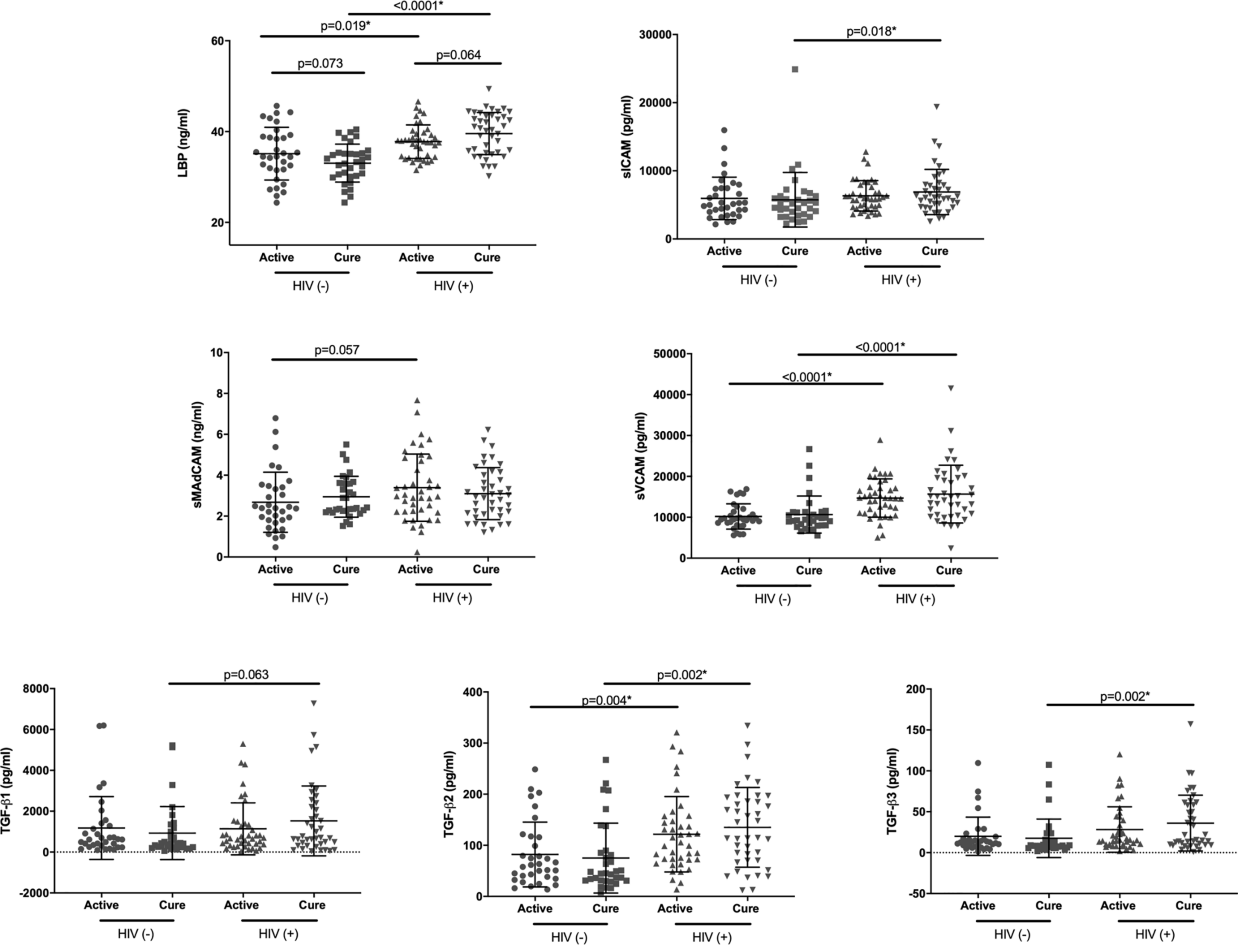
Plasma levels of LBP, sMAdCAM, sICAM, sVCAM, TGF-β1, TGF-β2 and TGF-β3 in HIV uninfected [HIV(-)] and HIV infected [HIV(+)] IMPRESS participants at active TB (active) and post- TB treatment (cure) timepoints. Based on the result of the normality test either parametric: paired t-test (Active vs Cure) and t-test (HIV+ vs HIV-), or non-parametric: Wilcoxon signed rank (Active vs Cure) and Mann-Whitney (HIV+ vs HIV-) tests were used for the analysis. *Statistically significant association after FDR adjustment using 0.05 threshold.

Next we examined the changes in plasma expression of measured analytes (LBP, sMAdCAM, sICAM, sVCAM, TGF-β1, TGF-β2 and TGF-β3) from active TB to post TB/cure in HIV infected (n= 38) and HIV uninfected (n = 33) participants from the IMPRESS study. We observed no significant differences in the expression of sMAdCAM, sICAM, sVCAM, TGF-β1, TGF-β2 and TGF-β3 between active TB and post TB cure in HIV infected and HIV uninfected participants (**Table 2**). A trend towards increased LBP levels following TB treatment was observed in HIV infected participants [Mean difference (MD) -2.03 (95% CI 4.17-0.12), p =0.064]. Conversely, HIV uninfected participants demonstrated the opposite trend, showing lower LBP levels at TB cure [MD 2.08 (95% CI -0.20-4.36), p =0.073] (**Figure 2**, **Table 2**).

**Table 2:** Changes in measured analytes in response to TB treatment in HIV infected (n = 38) and HIV uninfected (n = 33) IMPRESS study participants.

	HIV Infected (n = 38) Active TB – Post TB/Cure		HIV Uninfected (n = 33) Active TB – Post TB/Cure	
Variable	Mean Difference (95% CI)	p - value	Mean Difference (95% CI)	p - value
sMAdCAM	0.355 (-0.264 to 0.974)	0.252^a^	-0.264 (-0.75 to 0.221)	0.276^a^
sICAM	-905.385 (-1926.394 to 115.624)	0.193^b^	206.615 (-788.215 to 1201.446)	0.279^b^
sVCAM	-1200.169 (-3392.082 to 991.743)	0.633^b^	-485.88 (-2158.311 to 1186.552)	0.787^b^
LBP	-2.026 (-4.173 to 0.122)	0.064^a^	2.081 (-0.202 to 4.364)	0.073^a^
TGF-B1	-539.684 (-1190.915 to 131.547)	0.135^b^	246.483 (-466.95 to 959.917)	0.347^b^
TGF-B2	-18.832 (-51.919 to 14.255)	0.395^b^	7.006 (-21.552 to 35.564)	0.339^b^
TGF-B3	-10.189 (-23.778 to 3.399)	0.160^b^	2.42 (-9.019 to 13.858)	0.252^b^

a p – value is represented from the paired t-test.

b p – value is represented from the Wilcoxon signed rank test.

Additionally we examined the differences in expression levels of measured analytes between HIV uninfected and HIV infected participants at both active TB and TB cure timepoints. Expression of LBP, sVCAM and TGF-β2 were significantly elevated in HIV infected participants at both active TB and TB cure (p<0.02, **Figure 2**). Expression of sICAM (p=0.019) and TGF-β3 (p=0.002) were significantly elevated in HIV infected participants only at TB cure timepoint, likely due to TB – induced inflammatory changes at active TB (18). No significant differences were observed for sMADCAM and TGF-β1.

## Discussion

HIV-TB co-epidemic is one of the major health challenges affecting sub-Saharan Africa. Considering the geographical overlap of the HIV and TB epidemics and the 18 fold higher risk of TB in HIV co-infected (1), there is an urgent need to identify TB prognostic markers in HIV co-infected individuals, in order to improve patient management and fast-track the development of novel therapeutics. Here we identified increased levels of LBP and sICAM as biomarkers of risk of TB recurrence in HIV co-infected patients.

In the univariable and multivariable logistic models, the risk of TB recurrence was significantly associated with increase in plasma expression of sICAM and LBP, known to play a role in inflammation and translocation of microbial products, respectively (6, 19, 21). ICAM, present on endothelial cells, is involved in the firm arrest and transmigration of leukocytes from blood vessels to tissues, and is an important early marker of immune activation and response (7, 8, 22). Soluble, circulating forms of ICAM have been involved in a range of proinflammatory responses, and increase in sICAM levels have been linked with a range of human diseases including atherosclerosis and heart failure (21, 22). Previous studies have shown that the concentration of sICAM is elevated in patients with active TB disease (23, 24) and serum concentration of sICAM has been linked to bacterial burden (23, 25, 26) and disease severity (10). The observed increase in sICAM prior to TB disease likely reflects increased systemic inflammation as a result of increased bacterial replication from new infection or re-activation of latent disease.

In serum, LBP is present as a soluble acute-phase protein which binds to LPS and stimulates an immune response by presenting the LPS to cell surface pattern recognition receptors (PRRs) such as CD14 and toll like receptor 4 (TLR4) (20, 24). Increased concentrations of LBP have been observed in patients with sepsis and in healthy individuals injected with LPS (27, 28) as well as bronchoalveolar lavage fluids of heathy individuals and patients with lung injury (29). LBP was shown to be elevated in individuals with active TB and declined during treatment, suggesting that it may play an important role in the host reaction to TB (24). Increased levels of plasma LBP were associated with an increased risk of TB recurrence in this study. Since the TRuTH cohort only includes HIV co-infected individuals, increased LBP levels likely reflect both the HIV induced translocation of microbial products (30) as well as increase in TB bacterial burden. We also observed a strong positive correlation between LBP and sICAM as well as LBP and IL-6 identified to be one of the main pro-inflammatory cytokines associated with TB recurrence in our previous study (11). Interestingly, LPS was shown to be a strong inducer of both IL-6 and ICAM-1 (31, 32) suggesting that HIV-associated loss in gastrointestinal integrity and the resulting systemic inflammation could be fuelling the increased risk of TB recurrence in HIV co-infected individuals. HIV mediated gastrointestinal damage and the resulting translocation of microbial products (33) are known to cause immune activation and dysregulation of the host responses and as a result can lead to opportunistic infections including TB. This is supportive of a recent study in macaques co-infected with *M.tb* and SIV suggesting that SIV-driven chronic immune activation and dysregulation of T cell homeostasis associate with reactivation of latent TB (LTBI) (14). Furthermore changes in integrity of the gastrointestinal tract and resulting immune activation have been linked to lung inflammation, with 50% of adults who suffer with inflammatory bowel disease (IBD) and 33% of individuals who suffer with inflammatory bowel syndrome (IBS) having pulmonary involvement (34–36). This likely indicates that there is an inflammatory cross talk between different mucosal sites and that inflammation and dysbiosis in the gut can translate to inflammatory changes in the lungs (37).

After adjusting for covariates, the risk of TB recurrence was associated with increased concentrations of TGF-β3. TGF-β belongs to a superfamily of immunoregulatory cytokines which include three isoforms: TGF-β1, TGF-β2, and TGF-β3 (3). TGF-β controls the initiation and resolution of inflammation *via* regulation of chemotaxis, activation and survival of immune cells (38). Excess TGF-β was shown to supress T cell responses to *M.tb* antigens (39) and increased TGF-β activity is a feature of active pulmonary TB (40). Increase in TGF-β3 prior to TB recurrence likely reflects the increase in systemic inflammation. Additionally, TGF-β1 and TGF-β2 expression was positively correlated with sCD14 expression, a known marker of active TB (11, 18, 41, 42). While there is a general lack of isoform-specific data on TGF-β, isoform -specific knockout mice studies have shown non- redundant phenotypes. Specifically TGF-β3 knockout mice were shown to die perinatally due to developmental defects of the lung and defective palatogenesis (43).

We observed no significant changes in measured analytes between active TB disease and post TB treatment/cure time-points in a small subset of patients from TRuTH cohort. Likewise when we examined the changes in the expression of LBP, sMAdCAM, sICAM, sVCAM, TGF-β1, TGF-β2 and TGF-β3 from active TB disease to TB treatment completion in HIV infected and HIV uninfected individuals from IMPRESS, no significant differences were observed. Although not statistically significant, there was a trend towards decreased concentrations of plasma LBP in HIV uninfected individuals following TB treatment, likely due to a decrease in TB bacterial burden which is consistent with previous studies in predominantly HIV uninfected individuals (24). A trend towards increased concentrations of plasma LBP was observed in HIV infected individuals following TB treatment, likely reflecting a decrease in the gastrointestinal integrity caused by progressing HIV infection (33, 44). When we examined the differences in the relevant analytes between HIV infected and HIV uninfected individuals at active TB and post-successful TB treatment, we observed that LBP was significantly elevated in HIV infected individuals irrespective of the TB status, indicating that the HIV infection and resulting increase in systemic LPS exposure are the predominant drivers of increased LBP levels in co-infected patients. Plasma ICAM and TGF-β3 levels were only significantly elevated in HIV infected individuals post TB treatment completion, highlighting the effect of active TB in driving the increased expression of these markers (23, 25, 26).

Our study has several limitations. The observed associations are statistically modest and need to be confirmed in an independent human cohort to fully evaluate the predicative value of the identified biomarkers. The identified biomarkers of TB recurrence are only relevant in the context of TB-HIV co-infection. The analysis of the IMPRESS cohort is limited by an unequal gender distribution (specifically the predominance of men in the HIV-uninfected sample), a small sample size and resulting inability to stratify for potential confounders. Additional studies are needed to fully understand the relationship between treatment completion and disease severity, and the expression of the measured analytes.

In conclusion, we identified increased expression of plasma LBP and sICAM as biomarkers of TB recurrence in individuals who were receiving ART treatment in the TRuTH cohort. Our results support the notion of HIV associated chronic immune activation as the driving force in TB reactivation/reinfection in HIV infected individuals. Mitigating chronic immune activation through utilization of immune based interventions might not only lead to improved HIV outcome, but could significantly reduce the rates of TB recurrence and have a profound impact on reducing TB disease burden in HIV endemic settings. Further studies are needed to decipher the mechanism of how HIV mediated chronic immune activation increases the risk of TB reactivation in HIV co-infected individuals.

## Data Availability Statement

The datasets generated for this study are available on request to the corresponding author.

## Ethics Statement

The studies involving human participants were reviewed and approved by the University of KwaZulu-Natal’s Biomedical Research Ethics Committee (BE659/17). The patients/participants provided their written informed consent to participate in this study.

## Author Contributions

Designed the study: AS and KN. Performed the experiments: KP and AS. Analyzed the data: KP, AS, SR, LL, NY-Z, and DA. Wrote the first draft of the paper: KP and AS. Collected specimens and clinical data: SG, DG, and RH-M. Supervised clinical and/or experimental aspects of the study: AS, LM, DA, KN, NP, SA, and NS. All authors contributed to the article and approved the submitted version.

## Funding

The TRUTH study was supported by the Howard Hughes Medical Institute, Grant Number 55007065, as well as the Centers for Disease Control and Prevention (CDC) Cooperative Agreement Number UY2G/PS001350-02. Its contents are solely the responsibility of the authors and do not necessarily represent the official views of either the Howard Hughes Medical Institute or the Centers for Disease Control and Prevention (CDC). The research infrastructure to conduct this trial, including the data management, laboratory and pharmacy cores were established through the US National Institutes for Health’s Comprehensive International Program of Research on AIDS grant (CIPRA, grant # AI51794). AS is supported by European & Developing Countries Clinical Trials partnership (EDCTP) Career Development Fellowship (TMA2016CDF-1582). KP and SR was supported by the National Research Foundation (Grant numbers: 96354 and 108038 respectively). DA was supported the NRF Research Career Advancement Fellowship (grant # RCA13101656388) and a senior fellowship through the European and Developing Countries Clinical Trials Partnership (EDCTP) (grant # TMA2017SF-1960) funds. Any opinion, funding, and conclusion or recommendations expressed in this material is that of the author and the NRF does not accept liability in this regard. Patient care was supported by the KwaZulu-Natal Department of Health and the U.S. President’s Emergency Plan for AIDS Relief (PEPFAR). Research reported in this publication was supported by the Strategic Health Innovation Partnership (SHIP) Unit of the South African Medical Research Council, a grantee of the Bill & Melinda Gates Foundation, and the South African Medical Research Council. The funding sources listed here did not have any role in the analysis or preparation of the data in this manuscript, nor was any payment received by these or other funding sources for this manuscript.

## Conflict of Interest

The authors declare that the research was conducted in the absence of any commercial or financial relationships that could be construed as a potential conflict of interest.
