# Supplementary material for: Plasma Biomarkers of Risk of Tuberculosis Recurrence in HIV Co-Infected Patients From South Africa
Source: Front Immunol. 2021 Mar 25;12:631094. doi: 10.3389/fimmu.2021.631094 (PMC8026888; doi:10.3389/fimmu.2021.631094)
Supplement: Supplementary file 1 [file Table_1.docx]

**Supplemental Materials:**

**Supplement table 1.** Cohort characteristics of study participants from TRuTH

| **Variables** | **Recurrence of TB**  **(N = 37)** | **No Recurrence of TB**  **(N = 102)** | **p-value** |
| --- | --- | --- | --- |
| SAPiT enrolment |  |  |  |
| Randomization arm, n (%) |  |  |  |
| Early Arm | 12 (32.4) | 11 (10.8) | - |
| Post-Intensive Phase | 15 (40.5) | 39 (38.2) |  |
| Post-Continuation Phase | 10 (27) | 52 (51.0) |  |
| Age (y), median (IQR) | 32 (28 - 35) | 34 (28 - 40) | 0.025 |
| Body mass index (kg/m2) | 21.4 (19.6 - 23.1) | 21.8 (19.9 - 25.7) | 0.018 |
| CD4 count (cells/mm3), median (IQR) | 147 (73 - 234) | 145 (82 - 254) | 0.921 |
| Viral load (log copies/mL), mean (SD) | 5.1 (0.8) | 5.1 (0.8) | 0.593 |
| Gender, n (%) |  |  | 0.590 |
| Male | 19 (51.4) | 45 (44.1) |  |
| Female | 18 (48.6) | 57 (55.9) |  |
| WHO Stage, n (%) |  |  | 1.00 |
| 3 | 35 (94.6) | 95 (93.1) |  |
| 4 | 2 (5.4) | 7 (6.9) |  |
| Previous TB, n (%) |  |  | 0.441 |
| Yes | 13 (35.1) | 32 (31.4) |  |
| No | 24 (64.9) | 70 (68.6) |  |
| Lung cavities, n (%) |  |  | 0.666 |
| No | 19 (51.4) | 53 (52.0) |  |
| Yes | 14 (37.8) | 38 (37.3) |  |
| SAPiT ART Initiation |  |  |  |
| CD4 count (cells/mm^3^), median (IQR) | 145 (93 - 253) | 155 (93 - 259) | 0.920 |
| Viral load (log copies/ml), mean (SD) | 5.0 (0.8) | 5.0 (0.8) | 0.534 |
| End of TB treatment in SAPiT |  |  |  |
| CD4 count (cells/mm^3^), median (IQR) | 228 (139 - 446) | 191 (103 - 328) | 0.002 |
| Viral load (log copies/ml), mean (SD) | 3.0 (1.1) | 4.0 (1.4) | <0.001 |
| Variables at sample collection |  |  |  |
| Age (y), median (IQR) | 36 (33 - 39) | 39 (33 - 46) | 0.032 |
| CD4 count (cells/mm^3^), median (IQR) | 479 (339 - 834) | 459 (360 - 631) | 0.762 |
| Viral load (log copies/ml), mean (SD) | 1.6 (0.70) | 1.5 (0.6) | 0.319 |
| Abbreviation: ART, antiretroviral therapy; IQR, interquartile range; SD, standard deviation; TB, tuberculosis; WHO, World Health Organization | | | |

**Supplemental table 2.** Cohort characteristics of study participants from IMPRESS

| **Variables** | **HIV Infected (N=38)** | | **HIV Uninfected (N=33)** | |
| --- | --- | --- | --- | --- |
| ***Enrolment*** | | | | |
| Randomization arm, n (%) |  | |  | |
| HRZM – Active | 17 (44.7) | | 18 (54.6) | |
| HRZE – Control | 21 (55.3) | | 15 (45.4) | |
| Age at enrolment (years), [median (IQR)] | 35 (32-43) | | 33 (25–49) | |
| Gender, n (%) |  | |  | |
| Female | 16 (42.1) | | 2 (6.1) | |
| Male | 22 (57.9) | | 31 (93.9) | |
| # Previous TB episodes, n (%)^a^ |  | |  | |
| 1 | 32 (88.9) | | 28 (84.9) | |
| 2 | 4 (11.1) | | 3 (9.1) | |
| 3 | - | | 1 (3.0) | |
| 4 | - | | 1 (3.0) | |
| CD4 count (cells/mm^3^), median (IQR)^b^ | 233 (130 – 402) | | - | |
| Viral load (copies/mL), median (IQR) | 8,856 (<20-128,715) | | - | |
| ***Enrolment and follow-up*** | | | | |
|  | **Enrolment** | **Follow-up** | **Enrolment** | **Follow-up** |
| Body mass index (kg/m^2^) | 19.911 (18.416 – 23.054) | 21.336 (20.180-24.350) | 19.672 (18.327 – 21.565) | 21.673 (19.742-22.282) |
| Chest Findings, n (%)^c^ |  |  |  |  |
| Abnormal | 37 (100) | 30 (93.8) | 28 (100) | 27 (90) |
| Normal | 0 (0) | 2(6.2) | 0 (0) | 3 (10) |
| Cavities Right, n (%)^c^ |  |  |  |  |
| Yes | 15 (40.5) | 5 (15.6) | 17 (60.7) | 7 (23.3) |
| No | 22 (59.5) | 27 (84.4) | 11 (39.3) | 23 (76.7) |
| Cavities Left, n (%)^c^ |  |  |  |  |
| Yes | 14 (37.8) | 6 (18.8) | 16 (57.1) | 7 (23.3) |
| No | 23 (62.2) | 26 (81.2) | 12 (42.9) | 23 (76.7) |
|  |  |  |  |  |

Missing data: a= 2 missing number of previous TB episodes, b = 1 missing CD4 count at enrolment, c = 6 missing chest findings at enrolment, 9 at follow-up

Abbreviations: IQR, interquartile range; HRZE, Isoniazid, Rifampicin, Pyrazinamide, Ethambutol; HRZM, Isoniazid, Rifampicin, Pyrazinamide, Moxifloxacin; SD, standard deviation; TB, tuberculosis; WHO, World Health Organization

**Supplemental table 3.** Spearman correlation of sMAdCAM, sICAM, sVCAM, LBP, TGF-β1, TGF-β2 and TGF-β3 expression in TRuTH (n=139).

| **Cytokines/chemokines (N=)** | **sMAdCAM** | **sICAM** | **sVCAM** | **LBP** | **TGF-β1** | **TGF-β2** | **TGF-β3** |
| --- | --- | --- | --- | --- | --- | --- | --- |
| sMAdCAM |  | r= 0.173  **p= 0.042** | r=-0.026  p=0.760 | r=-0.073  p=0.392 | r=0.098  p=0.253 | r=0.099  p=0.246 | r=0.098  p=0.250 |
| sICAM | r= 0.173  **p= 0.042** |  | r=0.268  **p=0.01** | r= 0.347  **p=<0.001** | r=0.028  p=0.746 | r=-0.006  p=0.942 | r=-0.044  p=0.604 |
| sVCAM | r=-0.026  p=0.760 | r=0.268  **p=0.01** |  | r=0.049  p=0.569 | r=0.065  p=0.447 | r=0.020  p=0.817 | r=0.005  p=0.955 |
| LBP | r=-0.073  p=0.392 | r= 0.347  **p=<0.001** | r=0.049  p=0.569 |  | r=0.095  p=0.267 | r=-0.023  p=0.267 | r=0.032  p=0.708 |
| TGF-β1 | r=0.098  p=0.253 | r=0.028  p=0.746 | r=0.065  p=0.447 | r=0.095  p=0.267 |  | r=0.922  p=<**0.001** | r=0.847  **p=<0.001** |
| TGF-β2 | r=0.099  p=0.246 | r=-0.006  p=0.942 | r=0.020  p=0.817 | r=-0.023  p=0.267 | r=0.922  p=<**0.001** |  | r=0.846  **p=<0.001** |
| TGF-β3 | r=0.098  p=0.250 | r=-0.044  p=0.604 | r=0.005  p=0.955 | r=0.032  p=0.708 | r=0.847  **p=<0.001** | r=0.846  **p=<0.001** |  |

**Supplemental table 4.** Correlation of previously measured plasma cytokines/chemokines (Sivro et al. CID 2017) with the here measured analytes (sMAdCAM, sICAM, sVCAM, LBP, TGF-β1, TGF-β2 and TGF-β3) in matched samples from TRuTH.

| **Cytokines/chemokines (N=23)** | **sMAdCAM** | **sICAM** | **sVCAM** | **LBP** | **TGF-β1** | **TGF-β2** | **TGF-β3** |
| --- | --- | --- | --- | --- | --- | --- | --- |
| IL1β | r=-0.316  p=0.162 | r= -0.299  p= 0.188 | r=-0.057  p=0.806 | r=0.337  p=0.135 | r=0.123  p=0.594 | r=0.194  p=0.400 | r=0.305  p=0.179 |
| IL1Rα | r=-0.250  p=0.274 | r= -0.346  p= 0.124 | r=-0.049  p=0.832 | r=0.335  p=0.137 | r=0.068  p=0.769 | r=0.108  p=0.641 | r=0.220  p=0.338 |
| IL2 | r=0.234  p=0.308 | r= -0.348  p= 0.122 | r=0.216  p=0.346 | r=0.102  p=0.661 | r=-0.098  p=0.672 | r=-0.151  p=0.514 | r=-0.077  p=0.739 |
| IL-6 | r=-0.031  p=0.894 | r= 0.114  p=0.624 | r=0.264  p=0.248 | r=0.623  p=**0.003** | r=0.337  p=0.136 | r=0.438  **p=0.047** | r=0.321  p=0.156 |
| IL-7 | r=-0.134  p=0.563 | r=-0.342  p=0.130 | r=0.242  p=0.291 | r=0.273  p=0.232 | r=0.345  p=0.125 | r=0.481  p=**0.027** | r=0.231  p=0.314 |
| IL-10 | r=-0.227  p=0.322 | r=-0.252  p=0.271 | r=0.161  p=0.486 | r=0.387  p=0.083 | r=0.194  p=0.401 | r=0.286  p=0.209 | r=0.174  p=0.452 |
| IL-15 | r=0.369  p=0.099 | r=0.369  p=0.099 | r=0.258  p=0.258 | r=-0.148  p=0.523 | r=0.074  p=0.750 | r=0.000  p=1.000 | r=0.074  p=0.750 |
| IP10 | r=0.051  p=0.827 | r= -0.273  p= 0.232 | r=0.104  p=0.654 | r=0.264  p=0.248 | r=0.303  p=0.182 | r=0.353  p=0.117 | r=0.296  p=0.192 |
| TNF-α | r=-0.020  p=0.931 | r= 0.066  p= 0.775 | r=0.360  p=0.109 | r=0.338  p=0.134 | r=0.251  p=0.272 | r=0.254  p=0.266 | r=0.417  p=0.060 |
| IL1α | r=-0.057  p=0.807 | r= 0.049  p= 0.831 | r=0.105  p=0.651 | r=0.105  p=0.651 | r=-0.149  p=0.519 | r=-0.128  p=0.579 | r=-0.096  p=0.680 |
| IL-18 | r=-0.334  p=0.139 | r=-0.255  p=0.265 | r=-0.219  p=0.339 | r=0.117  p=0.614 | r=-0.010  p=0.964 | r=0.067  p=0.773 | r=0.141  p=0.542 |
| MIG | r=0.019  p=0.933 | r= 0.062  p=0.788 | r=-0.019  p=0.933 | r=0.131  p=0.571 | r=-0.145  p=0.529 | r=-0.012  p=0.960 | r=-0.180  p=0.435 |
| IFN-β | r=0.035  p=0.880 | r= -0.366  p=0.103 | r=-0.319  p=0.158 | r=-0.313  p=0.168 | r=-0.149  p=0.520 | r=-0.120  p=0.605 | r=-0.139  p=0.548 |
| IFN-λ2 | r=-0.316  p=0.162 | r=-0.035  p=0.881 | r=-0.280  p=0.219 | r=0.106  p=0.647 | r=-0.063  p=0.788 | r=0.052  p=0.822 | r=-0.109  p=0.638 |
| IFN-λ1 | r=0.148  p=0.523 | r=-0.074  p=0.750 | r=0.222  p=0.334 | r=-0.222  p=0.334 | r=0.111  p=0.633 | r=0.037  p=0.874 | r=0.037  p=0.874 |
| IFN-α2 | r=0.064  p=0.782 | r=0.118  p=0.611 | r=0.301  p=0.184 | r=0.023  p=0.921 | r=0.161  p=0.487 | r=0.231  p=0.313 | r=0.182  p=0.429 |
| IFN-γ | r=0.054  p=0.816 | r=0.412  p=0.064 | r=0.338  p=0.133 | r=-0.184  p=0.426 | r=-0.049  p=0.833 | r=-0.068  p=0.768 | r=0.047  p=0.840 |
| IL-8 | r=0.045  p=0.847 | r=-0.002  p=0.995 | r=0.007  p=0.976 | r=0.052  p=0.823 | r=-0.152  p=0.509 | r=-0.170  p=0.461 | r=-0.148  p=0.521 |
| IL12p40 | r=-0.265  p=0.245 | r=-0.356  p=0.113 | r=-0.248  p=0.278 | r=-0.092  p=0.691 | r=0.141  p=0.542 | r=0.162  p=0.483 | r=0.063  p=0.785 |
| IL12p70 | r=-0.243  p=0.289 | r=-0.071  p=0.760 | r=-0.156  p=0.500 | r=0.000  p=1.000 | r=-0.308  p=0.174 | r=-0.210  p=0.360 | r=-0.410  p=0.065 |
| IL-27 | r=0.258  p=0.258 | r=0.167  p=0.470 | r=0.438  **p=0.047** | r=0.141  p=0.542 | r=0.088  p=0.706 | r=-0.035  p=0.881 | r=0.284  p=0.213 |
| I-FAB | r=-0.013  p=0.956 | r=-0.065  p=0.778 | r=0.089  p=0.703 | r=-0.197  p=0.392 | r=0.275  p=0.228 | r=0.191  p=0.406 | r=0.200  p=0.385 |
| sCD14 | r=-0.212  p=0.357 | r=0.121  p=0.602 | r=0.179  p=0.437 | r=-0.250  p=0.915 | r=0.513  p=**0.017** | r=0.456  **p=0.038** | r=0.418  p=0.059 |

**Supplemental table 5.** Change in measured analytes in response to TB treatment in TRuTH (n=14).

| **TB – PostTB/Cure** | | | |
| --- | --- | --- | --- |
| **Variable** | **N** | **Mean difference (95% CI)** | **p-value** |
| **sMAdCAM** | 14 | -0.6479 (-1.452 to 0.156) | 0.105 |
| **sICAM** | 14 | 342.5 (-284.4 to 969.4) | 0.259 |
| **sVCAM** | 14 | 1375 (-881.3 to 3632) | 0.211 |
| **LBP** | 14 | -0.2771 (-1.416 to 0.8621) | 0.608 |
| **TGF-β1** | 14 | 7.641 (-94.58 to 109.9) | 0.874 |
| **TGF-β2** | 14 | -17.88 (-56.44 to 20.68) | 0.335 |
| **TGF-β3** | 14 | -16.4 (-56.62 to 23.82) | 0.394 |
